# Report of Last Fifty Operations for Cataract

**Published:** 1875-10

**Authors:** 


### REPORT OF FIFTY OPERATIONS FOR CATARACT.

| No. | NAME. | ADDRESS. | Age. | Dura'n | MODE of OPERATING. | RESULT. |
|---|---|---|---|---|---|---|
| 1 | S. D. Rouse, (Aunt.) | Upper Lisle, N. Y. | 70 | 20 | Upper Flap Extraction | Reads No. VI with x 2. |
| 2 | Mrs. Lucy H. Baldwin | Columbia X Roads, Pa. | 78 | 2 | " " " | " No. XX. |
| 3 | Mrs. Isaac Conover, | Flemingville, N. Y. | 60 | 2 | " " " | " No. VI with x 2½. |
| 4 | Walter Simonson, | Altay, N. Y. | 70 | 1 | Lower " " | Lost from suppurative infl'm'n |
| 5 | John E. Potter, | Williamsport, Pa. | 65 | 8 | Upper " " | Reads No. I with x 2½. |
| 6 | Mrs. Ezra Smith, | Jamestown, N. Y. | 60 | 12 | " " " | " No, XXX with x 5. |
| 7 | Mrs. Nicholas Dupert | Smithfield, Pa. | 63 | 4 | VonGraefe's Mo. Li. Ex. | " No. VI with x 2½. |
| 8 | A. C. Conn, | Williamsport, Pa. | 73 | 16 | " " | " No. VI with x 3. |
| 9 | David Flynn, | Rushville, N. Y. | 45 | 8 | " " | " No. XX with x 5. |
| 10 | John Goodman, | Hornellsville, N. Y. | 82 | 2 | Lower Flap Extract'n | " No. VI with x 2. |
| 11 | I. S. Rood, | Mott's Corners, N. Y. | 80 | 8 | Upper " " | " No. XX with x 5. |
| 12 | Wm. V. Havens, | Weedsport, N. Y. | 94 | 3 | Lower " " | " No. LX with x 5. |
| 13 | Mrs. Hanna Smith, | " " | 72 | 8 | Upper " " | " No. I with x 2½. |
| 14 | Levi Ballard, | Newark Valley, N. Y. | 60 | 1 | " " " | " No. VI (Twin brother operated upon 1 yr. ago, like result) |
| 15 | Mrs. Thomas, | Williamsport, Pa. | 65 | 1 | VonGraefe's Mo. Li Ex. | Reads No. VI with x 2. |
| 16 | Mrs. A. Tittsworth, | Bath, N. Y. | 70 | 2 | " " | " No. VI with x 2½. |
| 17 | Caleb Spaulding, | Memphis, N. Y. | 85 | 5 | Upper Flap Extract'n | " No. I with x 2½. |
| 18 | Mrs. Louisa Crebbs, | Warner, N. Y. | 82 | 4 | " " " | " No. VI with x 2. |
| 19 | Mrs. Sherburn, | " " | 71 | 4 | VonGraefe's Mo. Li. Ex. | " No. XX with x 5. |
| 20 | Stephen Loveland, | Windham, Brad. Co., Pa. | 65 | 2 | Upper Flap Extract'n | " No. XXX without glass's |
| 21 | Anson Abbey (Moth'r) | Weedsport, N. Y. | 72 | 4 | " " " | " No. I with x 2½. |
| 22 | Joseph Armstrong, | Newfield, Tomp. Co., N. Y. | 60 | 4 | Lower " " | " No. VI with x 2. |
| 23 | Hiram Rider, | Tioga, N. Y. | 65 | 1 | Upper " " | " No. VI with x 2½. |
| 24 | Wm. McRoy, | Syracuse, N. Y. | 54 | 2 | | " " " |
| 25 | Mrs. C. B. Paddock, | Bellisle, Onondaga Co., N. Y. | 60 | 2 | VonGraefe's Mo. Li Ex. | " " " |
| 26 | Mrs. C. E. Pendleton, | South Warren, Brad. Co., Pa. | 65 | 12 | " " | " " " |
| 27 | Patrick Kavenaugh. | Rathbonville, N. Y. | 45 | 3 | " " | " No. XX with x 2. |
| 28 | Mrs. Harriet Gould, | Port Byron, N. Y. | 60 | 4 | " " | " No. VI with x 2½. |
| 29 | Mrs. L. M. Patrick, | Buffalo, N. Y. | 68 | 6 | " " | " " 3 |
| 30 | John Fox, | Mainsburg, Pa. | 70 | 1 | " " | " " 2½ |
| 31 | ——Tubbs, | Binghamton, N. Y. | 35 | 2 | Upper Flap Extract'n | " No. XX with x 3. |
| 32 | Rockwell Hopper, | Bellfast, Catt. Co., N. Y. | 65 | 12 | " " " | Loss from suppurative infl'm'n |
| 33 | Martin Thomas, | Berkshire, Tioga Co., N. Y. | 60 | 4 | VonGraefe's Mo. Li Ex. | Reads No. VI with x 2½. |
| 34 | Horace Sunderlin, | Senne[illegible], N. Y. | 65 | 1 | Upper Flap Extract'n | Loss from suppurative infl'm'n |
| 35 | P. R. Creswell, | Alb[illegible]y, N. Y. | 62 | 3 | " " " | Reads No. VI with x 2. |
| 36 | Mrs. John Earhart, | Elmira, N, Y. | 63 | 4 | VonGraefe's Mo. Li Ex. | " No. XX with x 2½. |
| 37 | Mrs. A. H. Barnitz, | Coldwater, Mich. | 68 | 5 | " " | " No. VI with x 2½. |
| 38 | Mrs. Mary Harrington | Dryden, Tomp. Co., N. Y. | 83 | 2 | " " | " No. I with x 2½. |
| 39 | Mrs. A. Kellogg, | Jamaca, Vermont. | 72 | 8 | " " | " No. VI with x 2½. |
| 40 | Peter H. Conklin, | Brooklyn, N. Y. | 71 | 3 | " " | " " 2. |
| 41 | Samuel A. Hewett, | Genoa, Cayuga Co., N. Y. | 70 | 2 | " " | " " 2½. |
| 42 | Gardner Ballard, | Newark Valley, N. Y. | 60 | 3 | " " | " " 3. |
| 43 | John H. Baxter, | Decatur, Ill. | 70 | 6 | " " | " No. VI with x 2½. |
| 44 | Michael Murphy, | Ellicottville, N. Y. | 54 | 2 | Upper Flap Extract'n | " " " |
| 45 | James Tripp, | Dryden, Tompkins Co., N. Y. | 85 | 10 | " " " | " " " |
| 46 | M. W. Smith, (Moth'r) | South Warren, Pa. | 60 | 4 | VonGraefe's Mo. Li Ex. | " No. I with x 2½. |
| 47 | J. P. Jones, | " " | 75 | 5 | " " | " No. VI with x 2. |
| 48 | J. H. Beardsley, | Lyons, N. Y. | 74 | 5 | " " | " No. VI with x 2½. |
| 49 | E. O. Osgood, | Bradford, McKean Co., N. Y. | 51 | 33 | " " | " No. VI with x 2½ |
| 50 | D. Goulding, | Oswego, N. Y. | 73 | 5 | " " | Reads No. VI with x 2½. |

From the above table it will be discovered that out of fifty operations, *forty-seven* were restored to useful vision, six reading the finest print (No. I)—thirty-one, ordinary newspaper print (No. VI)—ten being able to read the larger letters, numbered from XX to LX, while three only were failures, through inflammation which destroyed the eye. Of the ten who saw only the largest letters, doubtless, most of them now can read No. VI, were a test to be made at the present time.

